# A Possibilistic Formulation of Autonomous Search for Targets

**DOI:** 10.3390/e26060520

**Published:** 2024-06-17

**Authors:** Zhijin Chen, Branko Ristic, Du Yong Kim

**Affiliations:** School of Engineering, RMIT University, 376-392 Swanston Street, Melbourne, VIC 3000, Australia; s3762807@student.rmit.edu.au (Z.C.); duyong.kim@rmit.edu.au (D.Y.K.)

**Keywords:** possibility theory, autonomous systems, robust estimation

## Abstract

Autonomous search is an ongoing cycle of sensing, statistical estimation, and motion control with the objective to find and localise targets in a designated search area. Traditionally, the theoretical framework for autonomous search combines sequential Bayesian estimation with information theoretic motion control. This paper formulates autonomous search in the framework of possibility theory. Although the possibilistic formulation is slightly more involved than the traditional method, it provides a means for quantitative modelling and reasoning in the presence of epistemic uncertainty. This feature is demonstrated in the paper in the context of partially known probability of detection, expressed as an interval value. The paper presents an elegant Bayes-like solution to sequential estimation, with the reward function for motion control defined to take into account the epistemic uncertainty. The advantages of the proposed search algorithm are demonstrated by numerical simulations.

## 1. Introduction

Search is a repetitive cycle of sensing, estimation (localisation), and motion control, with the objective to find and localise one, or as many as possible targets, inside the search volume, in the shortest possible time. The searching platform (agent) is assumed to be mobile and capable of sensing, while the detection process is typically imperfect [1], in the sense that the probability of detection is less than one, with a small (but non-negligible) probability of false alarms. Autonomous search refers to the search by an intelligent agent without human intervention.

Search techniques have been used in many situations. Examples include rescue and recovery operations, security operations (e.g., search for toxic or radioactive emissions), understanding animal behaviour, and military operations (e.g., anti-submarine warfare) [2]. Search techniques have also become increasingly important in field robotics [3,4,5,6] for the purpose of carrying out dirty and dangerous missions. A formal search theory has roots in the works by Koopman [7], and has since been expanded and extended to different problems and applications. It can be categorised into a static versus a moving target search, a reactive versus a non-reactive target search, a single versus multiple target search, a cooperative versus a non-cooperative target search, etc. [2].

In this paper, we focus on an area search for an unknown number of static targets using a realistic sensor on a single searching platform, conceptually similar to the problems discussed in [8,9,10]. The searching agent can be a drone equipped with a sensor capable of detecting targets on the ground with a certain probability of detection (as a function of range) as well with some false alarm probability.

The dominant theoretical framework for the formulation of search is probability theory, where Bayesian inference is used to sequentially update the posterior probability distribution of target locations as new measurements are collected over time [9,11,12,13,14]. Sensor motion control is typically formulated as a partially observed Markov decision process (POMDP) [15]. The information state in the POMDP formulation is represented by the posterior probability distribution of targets. The set of sensor motion controls (actions), which determine where the searching agent should move next, can be made a single step or multiple steps ahead. The reward function in POMDP maps the set of admissible actions to a set of positive real numbers (rewards) and is typically formulated as a measure of information gain (e.g., the reduction in entropy, Fisher information gain) [16].

Statistical inference is based on mathematical models. In the target search context, we need a model of sensing which incorporates the uncertainty with regards to the probability of true and false detection as well as the statistics of target (positional) measurement errors. This uncertainty in the Bayesian framework is expressed by probability functions—in particular, the probability of detection, the probability of false alarm, and the probability density function (PDF) of a positional measurement, given the true target location. The key limitation of the Bayesian approach, however, is that these probabilistic models must be known precisely. Unfortunately, in many practical situations it is difficult or even impossible to postulate precise probabilistic models. Consider, for example, the probability of detection. It typically depends on the (unknown) size and reflective characteristics of the target, and hence, at best can be specified as a confidence interval (rather than a precise probability value), for a given distance to the target. Thus, we need to deal with epistemic uncertainty, which incorporates both randomness and partial ignorance.

In order to deal with epistemic uncertainty, an alternative mathematical framework for inference is required. Such theories involve non-additive probabilities [17] for the representation and processing of uncertain information. They include, for example, possibility theory [18], Dempster–Shafer theory [19], and imprecise probability theory [20]. Because the last two theories are fairly complicated, and at present applicable only to discrete state spaces, we focus on possibility theory [21,22]. Recent research in nonlinear filtering and target tracking [23,24,25,26,27] has demonstrated that possibility theory provides an effective tool for uncertain knowledge representation and reasoning.

The main contributions of this paper include a theoretical formulation of autonomous search in the framework of possibility theory and a demonstration of its robustness in the presence of epistemic detection uncertainty. The paper presents an elegant Bayes-like solution to sequential estimation, with a definition of the reward function for motion control which takes into account the epistemic uncertainty. Evaluation of the proposed search algorithm considers scenarios with a large number of targets and for two cases for the probability of detection as a function of range: (i) the case when it is known precisely; (ii) the case when it is known only as an interval value.

The paper is organised as follows. Section 2 introduces the autonomous search problem. Section 3 reviews the standard probabilistic formulation of autonomous search and presents the theoretical framework for estimation using possibility functions. Section 4 formulates the new possibilistic solution to autonomous search. Numerical results with a comparison are presented in Section 5, while the conclusions are drawn in Section 6.

## 2. Problem Formulation

Consider a search area S. A surveillance drone, flying at a fixed altitude, has a mission to autonomously search and localise the ground-based static targets in S, as in [10]. The number and locations of the targets are unknown. Following [9,10], the search area S is discretized into $n_{c}\gg 1$ cells of equal size. The presence or absence of a target in the *n*th cell at a discrete time k=0,1,2,… can be modelled by a Bernoulli random variable (r.v.) $X_{k,n}\in \left\{0,1\right\}$, where by convention $X_{k,n}=1$ denotes that a target is present, (i.e., 0 denotes target absence) and $n=1,\ldots ,n_{c}$ is the cell index.

Suppose the search agent is equipped with a sensor (e.g., a radar) which illuminates a region $L_{k}\subset S$ at time *k* and collects a set of detections $Z_{k}$ within $L_{k}$. Each detection reports the Cartesian coordinates of a possible target. However, the sensing process is uncertain in two ways: (1) the reported target coordinates are affected by measurement noise; (2) the measurement set $Z_{k}$ may include false detections and also may miss some of the true target detections. The probability of true target detection is a (monotonically decreasing) function of range and is specified as an interval value for a given range.

The objective is to detect and localise as many targets as possible in the shortest possible time.

## 3. Background

### 3.1. Probabilistic Search

Autonomous search in the Bayesian probabilistic framework is typically information driven. The information state at time *k* is represented by the posterior probability of target *presence* in each cell of the discretised search area. This posterior probability at time *k* is denoted by $P_{k,n}=Pr\left\{X_{k,n}=1|Z_{1:k}\right\}$, where $Z_{1:k}:=Z_{1},\ldots ,Z_{k}$ is the sequence of measurement sets up to the current time *k*. The posterior probability of target *absence* is then simply ${\bar{P}}_{k,n}=Pr\left\{X_{k,n}=0|Z_{1:k}\right\}=1-P_{k,n}$, and therefore, is unnecessary to compute.

The target or threat map is defined as the array $P_{k}=\left[P_{k,n}\right]$. Initially, at time k=0, the map is specified as $P_{0,n}=\frac{1}{2}$, for all $n=1,\ldots ,n_{c}$, thus expressing the initial ignorance. As time progresses and the search agent collects measurements, the threat map is sequentially updated using Bayes’s rule. Consequently, the information content of the threat map $P_{k}$ increases with time. The information content of the threat map is measured by its entropy, defined as

(1) $$\begin{array}{c} H_{k}=-\frac{1}{n_{c}}\sum_{n=1}^{n_{c}}\left[P_{k,n}\log_{2}P_{n,k}+\left(1-P_{n,k}\right)\log_{2}\left(1-P_{n,k}\right)\right]. \end{array}$$

Note that at k=0, $H_{0}=1$ and that entropy decreases with time.

In order to explain how the threat map is updated using Bayes’s rule, let us introduce another Bernoulli r.v. $Y_{n,k}\in \left\{0,1\right\}$, where $Y_{n,k}=1$ represents the event that a detection from the set $Z_{k}$ has fallen inside the *n*th cell ($Y_{n,k}=0$ represents the opposite event). Bayes’s rule is given by

(2) $$Pr\left\{X=i|Y=j\right\}=\frac{Pr\{Y=j|X=i\}\;Pr\{X=i\}}{\sum_{\ell =0,1}Pr\left\{Y=j|X=\ell \right\}\;Pr\left\{X=\ell \right\}}$$

where the subscript (n,k) is temporarily removed from $X_{n,k}$ and $Y_{n,k}$ in order to simplify the notation, and i,j∈{0,1}.

Note that Pr{Y=1|X=1}=D and Pr{Y=1|X=0}=F represent the probability of detection and the probability of false alarm, respectively. Then, Pr{Y=0|X=1}=1−D and Pr{Y=0|X=0}=1−F.

Given $P_{k-1}$, if none of the detections in $Z_{k}$ falls into the nth cell (i.e., $Y_{n,k}=0$), the probability $P_{n,k}$ is updated according to (2), as

(3) $$P_{k,n}=\frac{\left(1-D_{k,n}\right)P_{k-1,n}}{\left(1-D_{k,n}\right)P_{k-1,n}+\left(1-F_{k,n}\right)\left(1-P_{k-1,n}\right)}$$

where $D_{k,n}$ is the probability of detection and $F_{k,n}$ is the probability of false alarm in the *n*th cell of search area S at time *k*.

If $Z_{k}$ contains a detection in the *n*th cell (i.e., $Y_{n,k}=1$), then the update equation according to (2) is

(4) $$P_{k,n}=\frac{D_{k,n}P_{k-1,n}}{D_{k,n}P_{k-1,n}+F_{k,n}\left(1-P_{k-1,n}\right)}$$

After collecting the measurement set $Z_{k-1}$, the searching agent must decide on its subsequent action, that is, where to move (and sense) next. Suppose the set of possible actions (for movement) is $A_{k}$. This set can be formed by considering one or more motion steps ahead (in the future). The reward function associated with every action $\alpha \in A_{k}$ is typically defined as the reduction in entropy of the threat map [10], that is,

(5) $$R_{k}\left(\alpha \right)=H_{k-1}-E\left\{H_{k}\left(\alpha \right)\right\}$$

Note the expectation operator E with respect to the (future) detection set $Z_{k}\left(\alpha \right)$. In practical implementation, in order to simplify computation, we typically adopt an approximation that circumvents E in (5). This approximation involves the assumption that a single realisation for $Z_{k}\left(\alpha \right)$ is sufficient: the one which results in hypothetical detection(s) at those cells which are characterised by a high probability of target presence, i.e., such that $P_{k-1,n}>\zeta$, where ζ is a threshold close to 1. The searching agent chooses the action which maximises the reward, i.e.,

(6) $$\alpha_{k}^{*}=\arg\max_{\alpha \in A_{k}}R_{k}\left(\alpha \right).$$

### 3.2. The Possibilistic Estimation Framework

Possibility theory is developed for quantitative modelling of epistemic uncertainty. The concept of the uncertain variable in possibility theory, plays the same role as the random variable in probability theory. The main difference is that the quantity of interest is not random, but simply unknown, and our aim is to infer its true value out of a set of possible values. The theoretical basis of this approach can be found in [28,29,30]. Briefly, the uncertain variable is a function X:Ω→X, where Ω is the sample space and X is the state space (the space where the quantity of interest lives). Our current knowledge about *X* can be encoded in a function $\pi_{X}:X\to \left[0,1\right]$, such that $\pi_{X}\left(x\right)$ is the possibility (credibility) for the event X=x. Function $\pi_{X}$ is not a density function, it is referred to as a possibility function, being the primitive object of possibility theory [22]. It can be viewed as a membership function determining the fuzzy restrictions of minimal specificity (in the sense that any hypothesis not known to be impossible cannot be ruled out) about *x* [18]. Normalisation of $\pi_{X}$ is $\sup_{x\in X}\pi_{X}\left(x\right)=1$ if X is uncountable, and $\max_{x\in X}\pi_{X}\left(x\right)=1$ if X is finite and countable.

In the formulation of the search problem, we will deal with two binary uncertain variables, corresponding to r.v.s $X_{k,n}$ and $Y_{k,n}$. Hence, let us focus on a discrete uncertain variable *X* and its state space $X=\{x_{1},\ldots ,x_{N}\}$. The possibility measure of an event A⊆X is a mapping $\Pi_{X}:2^{X}\to \left[0,1\right]$, where $2^{X}$ is the set of all subsets of X. Mapping $\Pi_{X}$ satisfies three axioms: (1) $\Pi_{X}\left(\emptyset \right)=0$; (2) $\Pi_{X}\left(X\right)=1$; and (3) the possibility of a union of disjoint events $A_{1}$ and $A_{2}$ is given by $\Pi_{X}\left(A_{1}\cup A_{2}\right)=\max\left[\Pi_{X}\left(A_{1}\right),\Pi_{X}\left(A_{2}\right)\right]$. Possibility measure $\Pi_{X}$ is related to the possibility function $\pi_{X}$ as follows:

$$\Pi_{X}\left(A\right)=\max_{x\in A}\pi_{X}\left(x\right)$$

for every A⊆X. There is also a notion of the *necessity* of an event $N_{X}\left(A\right)$, which is dual to $\Pi_{X}\left(A\right)$ in the sense that

(7) $$N_{X}\left(A\right)=1-\Pi_{X}\left(A^{c}\right),$$

where $A^{c}$ is the complement of *A* in X. One can interpret the necessity–possibility interval $\left[N_{X}\left(A\right),\Pi_{X}\left(A\right)\right]$ as the belief interval, specified by the lower and upper probabilities in the sense of Willey [20]. Note that for a binary variable X∈{0,1}, this interval can be expressed for event A={1} as $Pr\left\{X=1\right\}\in \left[N_{X}\left(1\right),\Pi_{X}\left(1\right)\right]=\left[1-\Pi_{X}\left(0\right),\Pi_{X}\left(1\right)\right]$, where, due to normalisation, the following condition must be satisfied: $\max\{\Pi_{X}\left(0\right),\Pi_{X}\left(1\right)\}=1$.

Bayes-like updating in possibility theory is described next. Suppose π(x) is the prior possibility function over the state space $X=\{x_{1},\ldots ,x_{N}\}$. Let γ(z|x) be the likelihood of receiving measurement z∈Z if x∈X is true. Then, the posterior possibility of x∈X is given by [28,31,32]

(8) $$\pi \left(x|z\right)=\frac{\gamma (z|x)\;\pi (x)}{\max_{x\in X}\left[\gamma \left(z|x\right)\;\pi \left(x\right)\right]}.$$

## 4. Theoretical Formulation of Possibilistic Search

### 4.1. Information State

The information state at time *k* in the framework of possibility theory will be represented by two posteriors:

1. The posterior possibility of target presence $\Pi_{k,n}^{1}=\Pi_{X_{k,n}}\left(\left\{1\right\}|Z_{1:k}\right)$;
2. The posterior probability of target absence $\Pi_{k,n}^{0}=\Pi_{X_{k,n}}\left(\left\{0\right\}|Z_{1:k}\right)$.

We need both of them, because $\Pi_{k,n}^{0}$ cannot be worked out from $\Pi_{k,n}^{1}$. Consequently, during the search two posterior possibility maps need to be updated sequentially over time, $\Pi_{k}^{1}=\left[\Pi_{k,n}^{1}\right]$ and $\Pi_{k}^{0}=\left[\Pi_{k,n}^{0}\right]$, where $n=1,\ldots ,n_{c}$.

Suppose now that the probability of detection is specified by an interval value, that is,

(9) $$D_{k,n}\in \left[{\underline{D}}_{k,n},{\bar{D}}_{k,n}\right]$$

where ${\underline{D}}_{k,n}$ and ${\bar{D}}_{k,n}$ represent the lower and upper probability of this interval, respectively. Because a detection event is a binary variable, due to the reachability constraint for probability intervals [33], (9) implies that the probability of non-detection is in interval $\left[1-{\bar{D}}_{k,n},1-{\underline{D}}_{k,n}\right]$. Then, via normalisation we can express the possibility of detection $D_{k,n}^{1}$ and the possibility of non-detection $D_{k,n}^{0}$ (in cell *n* at time *k*) as

(10) $$\begin{array}{cc} D_{k,n}^{1} & =\frac{{\bar{D}}_{k,n}}{\max\{1-{\underline{D}}_{k,n},{\bar{D}}_{k,n}\}} \end{array}$$

(11) $$\begin{array}{cc} D_{k,n}^{0} & =\frac{1-{\underline{D}}_{k,n}}{\max\{1-{\underline{D}}_{k,n},{\bar{D}}_{k,n}\}}. \end{array}$$

satisfying $\max\{D_{k,n}^{0},D_{k,n}^{1}\}=1$. Interval $\left[1-D_{k,n}^{0},D_{k,n}^{1}\right]$ represents the necessity–possibility interval for the probability of detection. Note that specification of a possibility function from a probability mass function expressed by probability intervals is not unique; for example, another more involved method for this task is via the maximum specificity criterion [34].

In general, the probability of detection $D_{k,n}$ by a sensor, as well as the two possibilities $D_{k,n}^{0}$ and $D_{k,n}^{1}$, are typically dependent on the distance $d_{n,k}$ between the *n*th grid cell and the searching agent’s position at time *k*.

In a similar manner, we can also assume that the probability of false alarm is specified by an interval value, that is, $F_{k,n}\in \left[1-F_{k,n}^{0},\;F_{k,n}^{1}\right]$, where $F_{k,n}^{0}$ and $F_{k,n}^{1}$ represent the possibility of no false alarm and the possibility of false alarm (in cell *n* at time *k*), respectively.

Next, we explain how to sequentially update, during the search, the two posterior possibility maps $\Pi_{k}^{1}$ (for target presence) and $\Pi_{k}^{0}$ (for target absence). The proposed update equations follow from (3) and (4), when we apply the Bayes-like update rule (8).

Given $\Pi_{k-1}^{1}$ and detection set $Z_{k}$, if none of the detections in $Z_{k}$ falls into the *n*th cell, the possibility of target presence in the *n*th cell is updated as follows:

(12) $$\Pi_{k,n}^{1}=\frac{D_{k,n}^{0}\;\Pi_{k-1,n}^{1}}{\max\{D_{k,n}^{0}\;\Pi_{k-1,n}^{1},\;F_{k,n}^{0}\;\Pi_{k-1,n}^{0}\}},$$

for $n=1,\ldots ,n_{c}$. Similarly, in this case $\Pi_{k,n}^{0}$ is updated according to

(13) $$\Pi_{k,n}^{0}=\frac{F_{k,n}^{0}\;\Pi_{k-1,n}^{0}}{\max\{D_{k,n}^{0}\;\Pi_{k-1,n}^{1},\;F_{k,n}^{0}\;\Pi_{k-1,n}^{0}\}}.$$

If a detection from $Z_{k}$ falls into the *n*th cell, then the update equation for $\Pi_{k,n}^{1}$ can be expressed as

(14) $$\Pi_{k,n}^{1}=\frac{D_{k,n}^{1}\;\Pi_{k-1,n}^{1}}{\max\{D_{k,n}^{1}\;\Pi_{k-1,n}^{1},\;F_{k,n}^{1}\;\Pi_{k-1,n}^{0}\}}.$$

And finally, in this case the update equation for $\Pi_{k,n}^{0}$ is given by

(15) $$\Pi_{k,n}^{0}=\frac{F_{k,n}^{1}\;\Pi_{k-1,n}^{0}}{\max\{D_{k,n}^{1}\;\Pi_{k-1,n}^{1},\;F_{k,n}^{1}\;\Pi_{k-1,n}^{0}\}}$$

Note that the probability of target presence in each cell of the search area, using the described possibilistic approach, is expressed by a necessity–possibility interval, i.e.,

(16) $$P_{k,n}\in \left[1-\Pi_{k,n}^{0},\;\Pi_{k,n}^{1}\right]$$

for $n=1,\ldots ,n_{c}$, where $\max\{\Pi_{k,n}^{0},\;\Pi_{k,n}^{1}\}=1$. Initially, at time k=0 (before any sensing action), the posterior possibility maps are set to

(17) $$\Pi_{0,n}^{0}=\Pi_{0,n}^{1}=1,$$

meaning that $P_{0,n}\in \left[0,1\right]$, for $n=1,\ldots ,n_{c}$. This is an expression of initial ignorance about the probability of target presence in the *n*th cell.

### 4.2. Epistemic Reward

Let us first quantify the amount of uncertainty contained in the information state, represented by two posterior possibility maps: $\Pi_{k}^{1}$ and $\Pi_{k}^{0}$. Various uncertainty (and information) measures in the context of non-additive probabilistic frameworks have been proposed in the past [35,36,37]. We adopt the principle that epistemic uncertainty corresponds to the volume under the possibility function [25,37]. For a possibility function π over a discrete finite state space $X=\{x_{1},\ldots ,x_{N}\}$, epistemic uncertainty equals the sum $\sum_{i=1}^{N}\pi \left(x_{i}\right)$. The possibilistic entropy $G_{k}$, contained in the information state, represented by $\Pi_{k}^{1}$ and $\Pi_{k}^{0}$, is then defined as

(18) $$\begin{array}{cc} G_{k}\; & =\frac{1}{n_{c}}\sum_{n=1}^{n_{c}}\left[\Pi_{k,n}^{1}+\Pi_{k,n}^{0}\right]-1 \end{array}$$

Equation (18) can be interpreted as the average volume of possibility functions of all binary variables $X_{n,k}$, for $n=1,\ldots ,n_{c}$. Subtraction by 1 on the right-hand side of (18) ensures that $G_{k}\in \left[0,1\right]$. Thus, at k=0, when $\Pi_{0,n}^{0}=\Pi_{0,n}^{1}=1$, we have $G_{0}=1$. This means that initially (at the start of the search), the amount of information contained in the information state is zero (representing total ignorance). As the searching agent moves and collects measurements it gains knowledge, and as a result either $\Pi_{k,n}^{0}$ or $\Pi_{k,n}^{1}$ will reduce its value in some cells (keeping in mind that $\max\{\Pi_{k,n}^{0},\;\Pi_{k,n}^{1}\}=1$), thus reducing the possibilistic entropy $G_{k}$. Finally, $G_{k}=0$ if either $\Pi_{k,n}^{0}=0$ (and due to normalisation $\Pi_{k,n}^{1}=1$) or $\Pi_{k,n}^{1}=0$ (and $\Pi_{k,n}^{0}=1$) for all cells $n=1,\ldots ,n_{c}$.

Note that (18) can also be expressed as

(19) $$\begin{array}{cc} G_{k} & =\frac{1}{n_{c}}\sum_{n=1}^{n_{c}}\left[\Pi_{k,n}^{1}-\left(1-\Pi_{k,n}^{0}\right)\right] \end{array}$$

which gives another interpretation of possibilistic entropy $G_{k}$: it represents the average necessity–possibility interval over all cells in the search area. This interpretation does not mean that $G_{k}$ is a measure of uncertainty only due to imprecision, because (18) and (19) are equivalent.

Similar to (5), we define the reward function as the reduction in possibilistic entropy of the information state, expressed by maps $\Pi_{k}^{1}$ and $\Pi_{k}^{0}$. Mathematically, this is expressed as

(20) $$R_{k}\left(\alpha \right)=G_{k-1}-E\left\{G_{k}\left(\alpha \right)\right\}$$

where, as before, $\alpha \in A_{k}$ is an action from the set of admissible actions at time *k* and E is the expectation with respect to the (random) measurement set $Z_{k}\left(\alpha \right)$. Again, in order to simplify the computation, we make the same assumption described in relation to (5): a single realisation for $Z_{k}\left(\alpha \right)$ consisting of hypothetical detection(s) at those cells which are characterised by $\Pi_{k-1,n}^{1}-\Pi_{k-1,n}^{0}>\zeta$. Finally, the searching agent chooses the action which maximises the reward, as in (6).

The search mission is terminated when the reduction in possibilistic entropy falls below a specified threshold, i.e., when $G_{k-1}-G_{k}<\xi$.

## 5. Numerical Results

### 5.1. Simulation Setup and a Single Run

**Figure 1:**
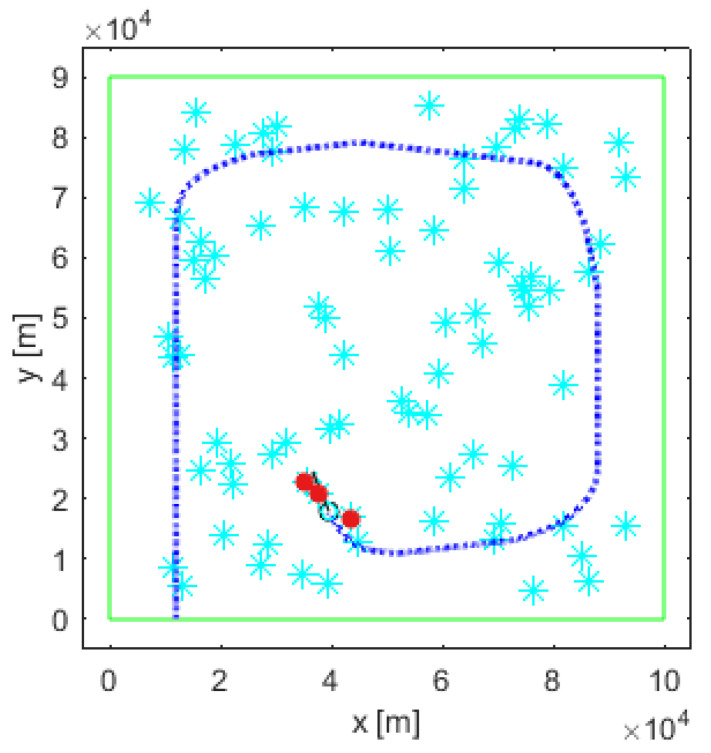
Simulation setup: the cyan stars indicate the true targets; the blue dotted line is the trajectory of the searching agent up to k=140 steps; the red dots indicate detections at k=140.

We use a simulation setup similar to [10]. The search area S is a rectangle of size 100 km × 90 km, discretised into $n_{c}=100\times 90$ resolution cells of size 1 km^2^. A total of 80 targets are placed at (a) uniformly random locations across the search area; (b) two squares in diagonal corners of the search area. A typical scenario with a uniform distribution of targets is shown in Figure 1, where cyan coloured asterisks indicate where the targets are placed.

**Figure 2:**
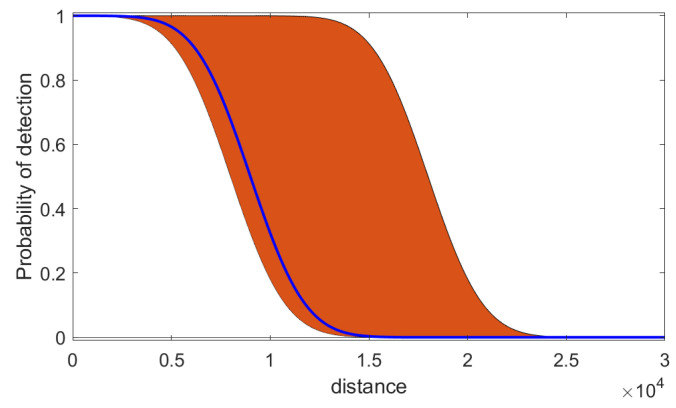
The imprecise model of the probability of detection *D* used in simulations. The true *D* is plotted with the blue solid line.

The probability of detection *D* is modelled as a function of the distance between the *n*th grid cell and the searching agent’s position at time *k*. The following mathematical model is adopted for this purpose:

(21) $$D\left(d;\mu ,\sigma \right)=1-\frac{1}{\sigma \sqrt{2\pi }}\int_{-\infty }^{d}e^{-\frac{{\left(t-\mu \right)}^{2}}{2\sigma^{2}}}dt$$

where d≥0 is the distance, while μ>0 and σ>0 are modelling parameters. Figure 2 illustrates this model; it displays the imprecise model of the probability of detection *D* as a function of distance *d*, using (21) with two sets of parameters μ and σ (the orange-coloured area). The search algorithm described in Section 4 is using this imprecise model for its search mission. The model provides the upper and lower probabilities ${\underline{D}}_{k,n}$ and ${\bar{D}}_{k,n}$ for a given range, from which we can work out $D_{k,n}^{1}$ and $D_{k,n}^{0}$, via (10) and (11), respectively. The true value of the probability of detection, which is used in the generation of simulated measurements (but which is unknown to the search algorithm), is plotted with the solid blue line in Figure 2. The truth is also based on model (21), using one particular pair of μ and σ values (The actual values used for the orange-coloured area in Figure 2 are $\mu_{1}=8000$, $\sigma_{1}=2200$, $\mu_{2}=$ 18,000, and $\sigma_{2}=2200$. The true probability of detection (blue line in Figure 2) is obtained using μ=9000 and σ=2200).

With this specification, the probability of detecting a target located more than a certain distance $\rho_{\max}$ from the searching agent is practically zero. Assuming $360^{\circ }$ coverage, the sensing area $L_{k}$ is a circular area of radius $\rho_{\max}$. The spatial distribution of false alarms is assumed to be uniform over $L_{k}$, with probability $F_{k,n}=0.005$ (per cell of $L_{k}$). For simplicity, we will assume that this parameter is known as the precise value to the search algorithm of Section 4. The threshold parameter ζ is set to 0.8.

Sensor measurements are affected by additive Gaussian noise with the standard deviation in range and azimuth of 100 m and $1^{\circ }$, respectively. An additional assumption is that there is at most one target per cell and one detection per cell.

The searching agent’s motion is modelled by the coordinated turn (CT) model [38], with the turning rate taking values from the set

Ψ={−0.4,−0.3,−0.2,−1,0,0.1,0.2,0.3,0.4}

(the units are °/s). We consider one-step ahead path planning, with action space $A_{k}$ defined as a Cartesian product $A_{k}=\Psi \times \Delta$. Here, Δ is the set of time intervals of CT motion (with the selected turning rate), adopted as Δ={60,120} seconds.

**Figure 3:**
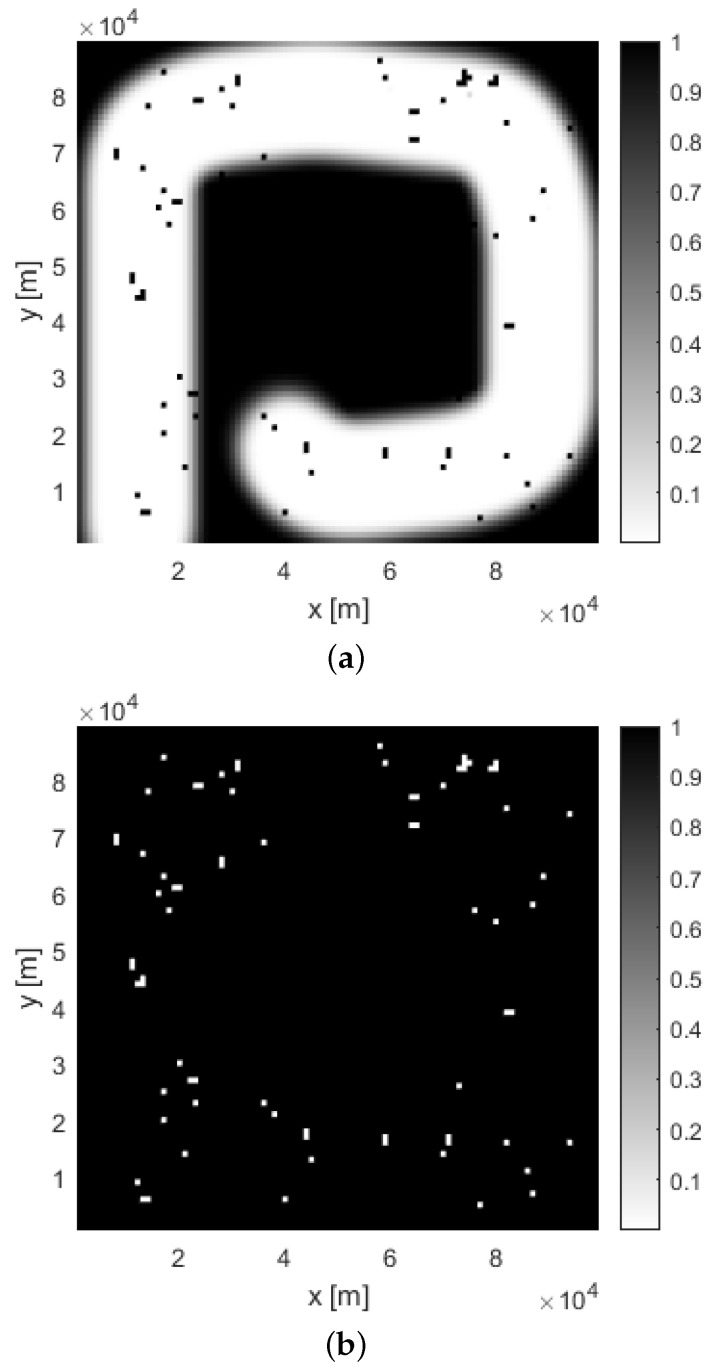
Posterior possibility maps at time k=140: (**a**) Target presence map $\Pi_{k}^{1}$; (**b**) target absence map $\Pi_{k}^{0}$ (white colour implies zero possibility).

**Figure 4:**
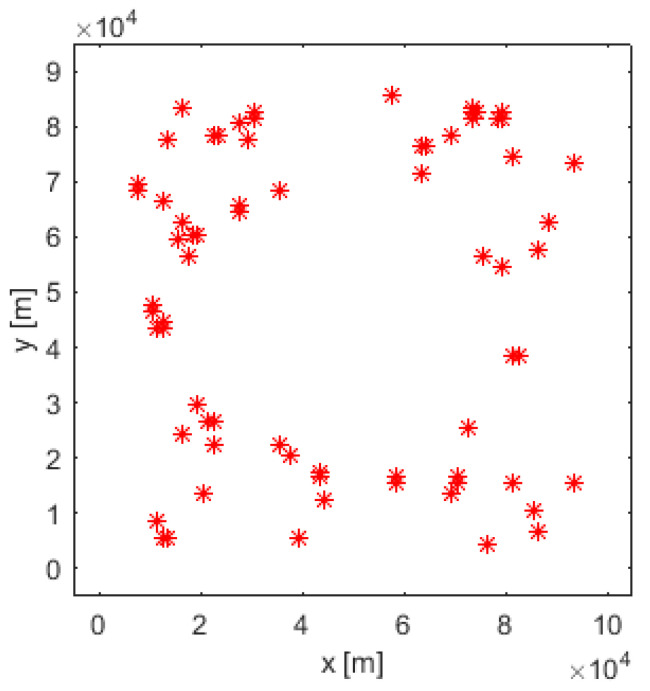
Output of the search algorithm: Estimated target locations (indicated by red asterisks).

The results of a single run of the possibilistic search at time k=140 for a uniform placement of targets is shown in Figure 1, Figure 3, and Figure 4. Figure 1 displays the search path (blue dotted line). The searching agent enters the search area S in the bottom left corner, and follows an inward-spiral path, in accordance with the probabilistic search [10]. Figure 3 shows the two posterior possibilistic maps: (a) target presence $\Pi_{k}^{1}$; and (b) target absence $\Pi_{k}^{0}$. The colour coding is as follows: white cells of the maps indicate zero possibility, while black cells denote the possibility is equal to 1. Figure 3a indicates that the area around the travelled path in $\Pi_{k}^{1}$ is mainly white, with occasional black cells where targets are possibly located. In those cells of the search area S where $\Pi_{k}^{1}$ is high (black colour) and $\Pi_{k}^{0}$ is low (white colour), there is a high chance that a target is placed. Therefore, the presence of a target in each cell of the search area is declared if the difference $\Pi_{k,n}^{1}-\Pi_{k,n}^{0}>0.8$.

The output of the search algorithm at k=140 is shown in Figure 4, which represents a map of estimated target positions: each red asterisk indicates a cell where the search algorithm declared a target. We can visually compare Figure 4 (estimated target positions at k=140) with Figure 1 (true target positions).

If the search were to be continued beyond k=140, the full spiral path would be completed at about k=200 (for an average run). After that, the rate of reduction in possibilistic entropy would significantly drop and the search algorithm would automatically stop (according to the termination criterion).

### 5.2. Monte Carlo Runs

Next, we compare the average search performance of the possibilistic search versus the probabilistic search. The adopted metric for search performance is the optimal sub-pattern assignment (OSPA) error, because it expresses in a mathematically rigorous manner the error both in the target position estimate and in the target number (cardinality error) [39]. The parameters of OSPA error used are cut-off c=50 km and order p=1. The mean OSPA error is estimated by averaging over 100 Monte Carlo runs, with a random placement of targets on every run. Because the search duration is random, for the sake of averaging the OSPA error, we fixed the duration to k=201 time steps.

In order to apply the probabilistic search for the problem specified in Section 5.1, we must adopt a precise (rather than an interval-valued) probability of detection. For comparison’s sake, we will consider two cases: (a) when the true probability of detection versus range (i.e., the blue line in Figure 2) is known; (b) given the interval-valued probability of detection (orange area in Figure 2), we choose the mid-point of the interval at a given range as the true value. Case (a) is ideal and is expected to result in the best performance, whereas case (b), because it uses an incorrect value of the probability of detection, is expected to perform worse.

**Figure 5:**
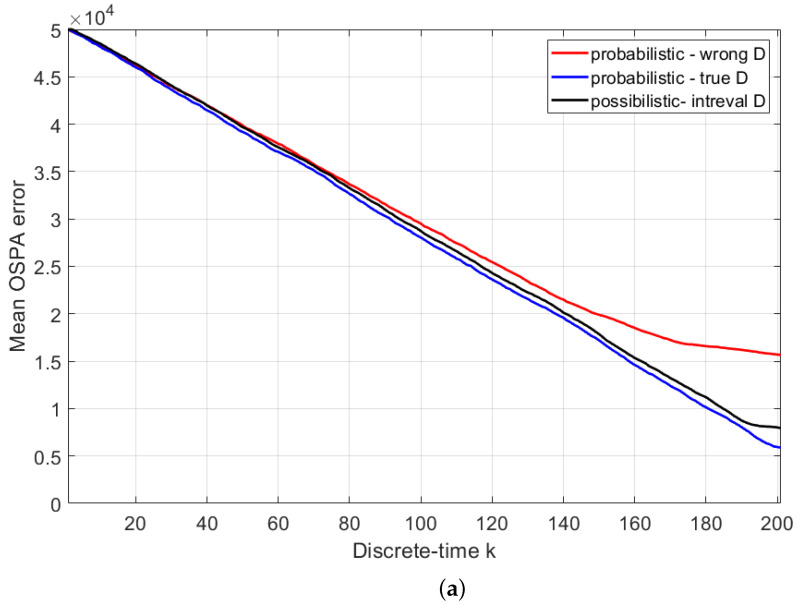

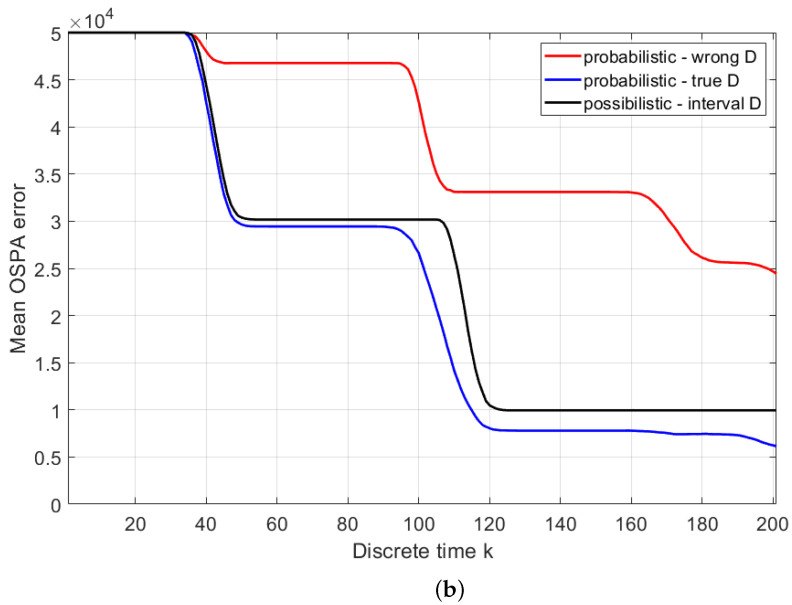
Mean OSPA errors obtained from 100 Monte Carlo runs. The scenario involves 80 targets placed at (**a**) uniformly random locations; (**b**) uniformly random locations of two diagonal squares in the search area.

The resulting three mean OSPA errors are presented in Figure 5 for two different target placements: (i) uniformly random target locations across the search area; (ii) random placement in two squares positioned in diagonal corners of the search area. The mean OSPA line colours in Figure 5 are as follows: black for possibilistic search; blue for probabilistic using true *D* (i.e., ideal case (a) above); red for probabilistic using wrong *D* (i.e., case (b)). All three mean OSPA error curves follow the same trend: they reduce steadily from the initial value of *c* as the searching agent traverses the area along the spiral path and discovers the targets. Of the three compared methods, as expected, the best performance (i.e., the smallest OSPA error) is achieved using the probabilistic with true *D* (ideal case). The possibilistic solution, which operates using the available interval-valued probability of detection, is fairly close to the ideal case. Finally, the probabilistic using the wrong value of *D* is the worst. The difference in performance is particularly dramatic when the placement of targets is non-uniform.

## 6. Conclusions

This paper formulated a solution to autonomous search for targets in the framework of possibility theory. The main rationale for the possibilistic formulation is its ability to deal with epistemic uncertainty, expressed by partially known probabilistic models. In this paper, we focused on the interval-valued probability of detection (as a function of range). The paper presented Bayes-like update equations for the information state in the possibilistic framework, as well as an epistemic reward function for motion control. The numerical results demonstrated that the proposed possibilistic formulation of search can deal effectively with epistemic uncertainty in the form of interval-valued probability of detection. As expected, the (conventional) probabilistic solution performs (sightly) better when the correct precise model of the probability of detection is known (the ideal model-match case). However, the probabilistic solution can result in dramatically worse performance if an incorrect precise model is adopted.

## Data Availability

Data is contained within the article.

## References

[1] Shlesinger M.F. (2006). Search research. Nature.

[2] Stone L.D., Royset J.O., Washburn A.R. (2016). Optimal Search for Moving Targets.

[3] Marjovi A., Marques L. (2011). Multi-robot olfactory search in structured environment. Robot. Auton. Syst..

[4] Pitre R.R., Li X.R., Delbalzo R. (2012). UAV route planning for joint search and track missions—An information-value approach. IEEE Trans. Aerosp. Electron. Syst..

[5] Burgués J., Marco S. (2020). Environmental chemical sensing using small drones: A review. Sci. Total Environ..

[6] Park M., An S., Seo J., Oh H. (2021). Autonomous source search for UAVs using Gaussian mixture model-based infotaxis: Algorithm and flight experiments. IEEE Trans. Aerosp. Electron. Syst..

[7] Koopman B.O. (1946). Search and screening. *OEG Rep*. https://www.loc.gov/resource/gdcmassbookdig.searchscreening56koop/?st=gallery.

[8] Bakut P.A., Zhulina Y.V. (1971). Optimal control of cell scanning sequence in search for objects. Eng. Cybern..

[9] Krout D.W., Fox W.L.J., El-Sharkawi M.A. (2009). Probability of target presence for multistatic sonar ping sequencing. IEEE J. Ocean. Eng..

[10] Angley D., Ristic B., Moran W., Himed B. (2019). Search for targets in a risky environment using multi-objective optimisation. IET Radar Sonar Navig..

[11] Kendall R.J., Presley S.M., Austin G.P., Smith P.N. (2008). Advances in Biological and Chemical Terrorism Countermeasures.

[12] Furukawa T., Mak L.C., Durrant-Whyte H., Madhavan R. (2012). Autonomous Bayesian search and tracking, and its experimental validation. Adv. Robot..

[13] Ristic B., Skvortsov A., Gunatilaka A. (2016). A study of cognitive strategies for an autonomous search. Inf. Fusion.

[14] Haley K. (2012). Search Theory and Applications.

[15] Chong E.K.P., Kreucher C.M., Hero A.O. (2008). POMDP approximation using simulation and heuristics. Foundations and Applications of Sensor Management.

[16] Hero A.O., Kreucher C.M., Blatt D. (2008). Information theoretic approaches to sensor management. Foundations and Applications of Sensor Management.

[17] Hampel F. (2009). Nonadditive probabilities in statistics. J. Stat. Theory Pract..

[18] Zadeh L.A. (1978). Fuzzy sets as a basis for a theory of possibility. Fuzzy Sets Syst..

[19] Yager R.R., Liu L. (2008). Classic Works of the Dempster-Shafer Theory of Belief Functions.

[20] Walley P. (1991). Statistical Reasoning with Imprecise Probabilities.

[21] Dubois D., Prade H., Sandri S. (1993). On possibility/probability transformations. Fuzzy Logic: State of the Art.

[22] Dubois D., Prade H. (2015). Possibility theory and its applications: Where do we stand?. Springer Handbook of Computational Intelligence.

[23] Ristic B., Houssineau J., Arulampalam S. (2019). Robust target motion analysis using the possibility particle filter. IET Radar Sonar Navig..

[24] Ristic B., Houssineau J., Arulampalam S. (2020). Target tracking in the framework of possibility theory: The possibilistic Bernoulli filter. Inf. Fusion.

[25] Chen Z., Ristic B., Houssineau J., Kim D.Y. (2021). Observer control for bearings-only tracking using possibility functions. Automatica.

[26] Houssineau J., Zeng J., Jasra A. (2021). Uncertainty modelling and computational aspects of data association. Stat. Comput..

[27] Ristic B. (2023). Target tracking in the framework of possibility theory. ISIF Perspect. Inf. Fusion.

[28] Houssineau J., Bishop A.N. (2018). Smoothing and filtering with a class of outer measures. SIAM/ASA J. Uncertain. Quantif..

[29] Bishop A.N., Houssineau J., Angley D., Ristic B. (2018). Spatio-temporal tracking from natural language statements using outer probability theory. Inf. Sci..

[30] Houssineau J. (2021). A linear algorithm for multi-target tracking in the context of possibility theory. IEEE Trans. Signal Process..

[31] Boughanem M., Brini A., Dubois D. (2009). Possibilistic networks for information retrieval. Int. J. Approx. Reason..

[32] Ristic B., Gilliam C., Byrne M., Benavoli A. (2020). A tutorial on uncertainty modeling for machine reasoning. Inf. Fusion.

[33] Campos L.M.D., Huete J.F., Moral S. (1994). Probability intervals: A tool for uncertain reasoning. Intern. J. Uncertain. Fuzziness-Knowl.-Based Syst..

[34] Masson M.-H., Denoeux T. (2006). Inferring a possibility distribution from empirical data. Fuzzy Sets Syst..

[35] Klir G.J., Smith R.M. (2001). On measuring uncertainty and uncertainty-based information: Recent developments. Ann. Math. Artif. Intell..

[36] Abellán J. (2006). Uncertainty measures on probability intervals from the imprecise dirichlet model. Intern. J. Gen. Syst..

[37] Pota M., Esposito M., Pietro G.D. (2013). Transforming probability distributions into membership functions of fuzzy classes: A hypothesis test approach. Fuzzy Sets Syst..

[38] Bar-Shalom Y., Li X.R., Kirubarajan T. (2004). Estimation with Applications to Tracking and Navigation: Theory Algorithms and Software.

[39] Schuhmacher D., Vo B.-T., Vo B.-N. (2008). A consistent metric for performance evaluation of multi-object filters. IEEE Trans. Signal Process..

